# Supplementary material for: Semi-parametric empirical bayes method for multiplet detection in snATAC-seq with probabilistic multi-omic integration
Source: PLoS Comput Biol. 2026 Apr 29;22(4):e1013653. doi: 10.1371/journal.pcbi.1013653 (PMC13148828; doi:10.1371/journal.pcbi.1013653)
Supplement: S1 Text — (PDF) [file pcbi.1013653.s001.pdf]

Supplementary Methods and Results

1 Negative Binomial Mixture Model

We implement a negative bionomial mixture model for comparison. Here, we provide the relevant technical details.

1.1 Modeling Assumption

We focus exclusively on detecting doublets, as they account for more than 97% of multiplets in droplet-based single-cell assays when the overall multiplet rate is below 5% and complete cell dissociation is achieved [1]. Although the approach can be extended to higher-order multiplets, their rarity under these conditions makes such modeling unnecessary for most datasets. Also in our simulation benchmarking, we simulate only doublets (i.e., pairs of cells) and do not generate higher-order multiplets; accordingly, the model includes a doublet component but not components for triplets or larger aggregates.

Let $\boldsymbol{X} = x_1, \ldots, x_N$ denote the HCLC values for N cell barcodes in an experiment. We model the empirical distribution of $\boldsymbol{X}$ using a two-component mixture of Negative Binomial (NB) distributions to differentiate between singlets and doublets:

$$f(x_j; \theta) = \pi_0 \cdot \mathrm{NB}(x_j; r, p) + (1 - \pi_0) \cdot \mathrm{NB}(x_j; 2r, p), \tag{S1}$$

where π_0 is the proportion of singlets among the retained cells, r is the NB size parameter (dispersion), and p is the NB success probability. The singlet density corresponds to $\mathrm{NB}(x_j; r, p)$, while the doublet density is modeled as $\mathrm{NB}(x_j; 2r, p)$, based on the assumption that a doublet can be approximated as the sum of two independent singlets. The vector of all unknown parameters in the model is $\boldsymbol{\theta} = (\pi_0, r, p)$.

1.2 Parameter Estimation

We estimate the parameters $\boldsymbol{\theta} = (\pi_0, r, p)$ in Equation S1 using the Expectation–Maximization (EM) algorithm. Let $z_j \in \{1, 2\}$ be the unobserved indicator for the class label of cell j, where $z_j = 1$ denotes a singlet and $z_j = 2$ denotes a doublet. The complete-data log-likelihood is given by

$$\log L(\boldsymbol{\theta} \mid \boldsymbol{X}, \mathbf{z}) = \sum_{j=1}^{N} \sum_{k=1}^{2} I(z_j = k) \left[\log \pi_k + \log \mathrm{NB}(x_j; r_k, p)\right],$$

where $\pi_1 = \pi_0$, $\pi_2 = 1 - \pi_0$, $r_1 = r$, and $r_2 = 2r$.

The EM algorithm alternates between the following two steps until convergence:

E-step. With current parameters $\boldsymbol{\theta}^{(m)} = (\pi_0^{(m)}, r^{(m)}, p^{(m)})$, compute the posterior class probabilities

$$\gamma_{jk}^{(m)} = P(z_j = k \mid x_j, \boldsymbol{\theta}^{(m)}) = \frac{\pi_k^{(m)} \mathrm{NB}(x_j; r_k^{(m)}, p^{(m)})}{\sum_{\ell=1}^{2} \pi_\ell^{(m)} \mathrm{NB}(x_j; r_\ell^{(m)}, p^{(m)})}, \quad k \in \{1, 2\},$$

and the incomplete log-likelihood

$$\ell_{\mathrm{inc}}^{(m)} = \sum_{j=1}^{N} \log \left[\sum_{k=1}^{2} \pi_k^{(m)} \mathrm{NB}(x_j; r_k^{(m)}, p^{(m)}) \right].$$

M-step.

1. Update the singlet proportion:

$$\pi_0^{(m+1)} = \frac{\sum_{i=1}^{N} \gamma_{i1}^{(m)}}{\sum_{i=1}^{N} \gamma_{i1}^{(m)} + \sum_{i=1}^{N} \gamma_{i2}^{(m)}}.$$

2. Update the size parameter r by solving the score equation with p fixed at $p^{(m)}$:

$$0 = \sum_{i=1}^{N} P(z_j = 1 \mid x_j, \boldsymbol{\theta}^{(m)}) \left[\psi(x_j + r) - \psi(r) + \log p^{(m)}\right]$$
$$+ \sum_{i=1}^{N} P(z_j = 2 \mid x_j, \boldsymbol{\theta}^{(m)}) \left[2\psi(x_j + 2r) - 2\psi(2r) + 2\log p^{(m)}\right],$$

where $\psi(\cdot)$ is the digamma function. The root is obtained using the Newton–Raphson method.

3. Update the probability parameter p using the new $r^{(m+1)}$:

$$p^{(m+1)} = \frac{r^{(m+1)}\big(\sum_{i=1}^{N} \gamma_{i1}^{(m)} + 2\sum_{i=1}^{N} \gamma_{i2}^{(m)}\big)}{\sum_{i=1}^{N} x_i\gamma_{i1}^{(m)} + \sum_{i=1}^{N} x_i\gamma_{i2}^{(m)} + r^{(m+1)} \sum_{i=1}^{N} \big(\gamma_{i1}^{(m)} + 2\gamma_{i2}^{(m)}\big)}.$$

Convergence criterion. The algorithm terminates when the absolute change in the incomplete log-likelihood between successive iterations is less than a predefined threshold ϵ (here, $\epsilon = 10^{-8}$), or when a maximum of 5000 iterations is reached.

1.3 Modeling doublets in Negative Binomial Model

Here, we show that doublets follow NB$(2r, p)$ in our negative binomial modeling framework.

Moment generating function of NB(r, p). For $X \sim \text{NB}(r, p)$ with support $x \geq r$,

$$\begin{aligned}
M_X(t) = \mathbb{E}\big[e^{tX}\big] &= \sum_{x=r}^{\infty} e^{tx} \binom{x-1}{r-1} (1-p)^{x-r} p^r \\
&= p^r e^{tr} \sum_{x=r}^{\infty} \binom{x-1}{r-1} \big[(1-p)e^t\big]^{x-r} \\
&= (pe^t)^r \sum_{k=0}^{\infty} \binom{k+r-1}{r-1} \big[(1-p)e^t\big]^k \quad (k = x - r) \\
&= (pe^t)^r \left[1 - (1-p)e^t\right]^{-r} \\
&= \left[\frac{pe^t}{1-(1-p)e^t}\right]^r.
\end{aligned}$$

Distribution of a sum of independent NB variables. Let $X_1, X_2, \ldots, X_n$ be independent with $X_i \sim \mathrm{NB}(r_i, p)$. For

$$Y = \sum_{i=1}^{n} X_i,$$

the MGF factorizes:

$$\begin{aligned} M_Y(t) = \mathbb{E}\left[e^{tY}\right] = \prod_{i=1}^{n} \mathbb{E}\left[e^{tX_i}\right] &= \prod_{i=1}^{n} \left[\frac{pe^t}{1-(1-p)e^t}\right]^{r_i} \\ &= \left[\frac{pe^t}{1-(1-p)e^t}\right]^{\sum_{i=1}^{n} r_i}. \end{aligned}$$

Hence,

$$Y \sim \mathrm{NB}\left(\sum_{i=1}^{n} r_i,\ p\right).$$

Special case: doublets. A doublet is modeled as the sum of two independent singlets, $X_{\mathrm{dbl}} = X_1 + X_2$, where $X_1, X_2 \overset{\text{i.i.d.}}{\sim} \mathrm{NB}(r, p)$. By the above result,

$$X_{\mathrm{dbl}} \sim \mathrm{NB}(2r, p),$$

justifying the use of $\mathrm{NB}(2r, p)$ for the doublet component in Equation S1.

2 FDR to local fdr Conversion

In the SEBULA framework, we initially estimate a tail-area False Discovery Rate (FDR) for multiplet classification. However, for downstream probabilistic multi-omic integration, we require the local false discovery rate (local fdr), which represents the posterior probability that a given cell belongs to the singlet population.

Because the tail-area FDR is mathematically equivalent to the expectation (or average) of the local fdr values within that tail region, we can empirically recover the local fdr from the FDR estimates using a discrete differencing approach, followed by an optional isotonic regression to ensure statistical consistency. The conversion algorithm proceeds through the following steps:

Rank-Based Ordering and Cumulative Expected Errors We first sort all evaluated cells in ascending order based on their computed FDR values. Let k represent the rank of a cell (from 1 to n), and FDR_k represent its corresponding tail-area FDR. Because the FDR at rank k is the average of the local fdr values for all cells up to that rank, the cumulative expected number of false discoveries (true singlets falsely classified as multiplets) among the top k cells is simply calculated as $k \times \text{FDR}_k$.

Discrete Differencing To isolate the local fdr for the k-th cell, we calculate the marginal change in the expected number of false discoveries. We take the discrete difference between rank k and rank $k-1$:

$$\text{lfdr}_k = (k \times \text{FDR}_k) - ((k-1) \times \text{FDR}_{k-1})$$

For the top-ranked cell ($k = 1$), the local fdr is set directly to FDR_1.

Boundary Enforcement Because empirical discrete differencing can occasionally produce numerical artifacts or overshoot valid probability boundaries, we clip the raw local fdr estimates to ensure they strictly fall within the $[0, 1]$ interval.

Monotonicity Smoothing via Isotonic Regression: Theoretically, as the tail-area FDR and cell rank increase (indicating decreasing confidence that the cell is a multiplet), the local fdr must monotonically increase. However, raw empirical differencing often yields a noisy, non-monotonic

sequence. To improve stability, we optionally apply isotonic regression smoothing to the recovered lfdr sequence. This step enforces a monotonic relationship between the ranking of cells and their estimated singlet probabilities, which is consistent with the assumption that stronger multiplet evidence should not correspond to higher singlet probability. The isotonic regression is performed on the ordered lfdr values and preserves their overall structure while reducing local fluctuations. After these steps, the smoothed local fdr values are mapped back to their original cell barcodes. These values can then be directly converted into multiplet posterior probabilities ($1 - \text{lfdr}$) for use in SEBULA's Bayesian integration module.

3 Details of Bayesian Integration Evidence for Doublet Detection

3.1 Integrating Evidence from Classification Probability

To integrate complementary information across modalities for doublet classification, we adopt a probabilistic framework for estimating the posterior probability that a given cell is a singlet. Let X_1 denote the RNA-derived feature vector used to train a classifier (e.g., scDblFinder), and let X_2 represent a chromatin-based scalar signal HCLC.

From the RNA modality, we obtain a singlet probability score $p_1 = \Pr(\text{Singlet} \mid X_1) \in (0, 1)$, derived from the classifier output (e.g., scDblFinder score) and interpreted as evidence for singlet status based on transcriptional features alone. Although this quantity is not necessarily a fully calibrated Bayesian posterior under the true biological prior, it is monotonic with singlet likelihood and can be treated as a modality-specific probability estimate for integration.

We then incorporate additional information from the ATAC-derived signal using Bayes' theorem. Under conditional independence of X_1 and X_2 given the class label, the posterior probability can be written as

$$\Pr(\text{Singlet} \mid X_1, X_2) \propto \Pr(X_2 \mid \text{Singlet}) \cdot \Pr(\text{Singlet} \mid X_1).$$

Here, $\Pr(X_2 \mid \text{Singlet})$ denotes the likelihood of observing the chromatin signal under the singlet population. In our framework, this likelihood is derived from a two-component mixture model applied to X_2. Let $f(X_2)$ denote the empirical marginal density of the signal and $f_0(X_2)$ the estimated density under singlets. The local false discovery rate is defined as

$$\text{lfdr}(X_2) = \Pr(\text{Singlet} \mid X_2) = \frac{\pi_0 f_0(X_2)}{f(X_2)},$$

where π_0 is the estimated prior proportion of singlets.

Rearranging this expression yields

$$\frac{\Pr(X_2 \mid \text{Singlet})}{\Pr(X_2)} = \frac{f_0(X_2)}{f(X_2)} = \frac{\text{lfdr}(X_2)}{\pi_0}.$$

Substituting into the posterior update gives

$$\Pr(\text{Singlet} \mid X_1, X_2) \propto \frac{\text{lfdr}(X_2)}{\pi_0} \cdot \Pr(\text{Singlet} \mid X_1).$$

3.1.1 Generalized Multi-Source Integration via Bayes Factors

The above formulation naturally extends to settings where multiple sources of evidence $X_1, X_2, \ldots, X_K$ are available. Let $Y \in \{\text{Singlet}, \text{Doublet}\}$ denote the latent class label. We assume conditional independence of the observed features given Y.

For each modality k, let $p_k \in (0, 1)$ denote a singlet probability score derived from that modality. These scores may represent posterior probabilities under a modality-specific model (e.g., lfdr from a mixture model) or classifier-derived probability estimates (e.g., logistic outputs from discriminative models). While such quantities may not be perfectly calibrated under the true biological prior, they provide monotonic evidence for singlet status.

Let π denote the global prior proportion of singlets in the dataset. Under Bayes' theorem, the Bayes factor contributed by modality k can be expressed as

$$\text{BF}_k(X_k) = \frac{\Pr(X_k \mid \text{Singlet})}{\Pr(X_k \mid \text{Doublet})} = \frac{p_k(1-\pi)}{(1-p_k)\pi}.$$

Starting from the baseline posterior odds derived from one modality (e.g., RNA),

$$\text{Odds}_1 = \frac{p_1}{1-p_1},$$

we sequentially update the odds by multiplying the Bayes factors from additional modalities:

$$\text{Odds}_{1:K} = \text{Odds}_1 \cdot \prod_{k=2}^{K} \text{BF}_k.$$

The integrated posterior probability is then obtained via the standard odds-to-probability transformation:

$$\Pr(\text{Singlet} \mid X_1, \ldots, X_K) = \frac{\text{Odds}_{1:K}}{1 + \text{Odds}_{1:K}}.$$

This sequential updating framework provides a modular and extensible mechanism for integrating heterogeneous sources of evidence. Under conditional independence, each modality contributes multiplicative evidence through its Bayes factor. In practice, moderate dependence between modalities primarily affects probability calibration rather than classification ranking, and therefore does not materially alter decision boundaries.

3.2 Integrating Evidence from p-values or z-scores

While SEBULA natively provides posterior probabilities based on the HCLC statistic, some existing multiplet detection tools and external modalities report evidence in the form of frequentist p-values or z-scores. To seamlessly incorporate these signals into our probabilistic framework, we map these frequentist metrics into a Bayesian evidence scale.

For a method that outputs a p-value for a given droplet (denoted as evidence y), we first convert it to a corresponding z-score. We then square this value to obtain z^2, which serves as the foundation for computing the Approximate Bayes Factor (ABF) based on Wakefield's method [2].

Wakefield's ABF provides a robust approximation of the Bayes factor comparing the alternative hypothesis (H_1: the droplet is a multiplet) to the null hypothesis (H_0: the droplet is a singlet). Assuming a standardized variance for the z-score, the ABF in favor of the multiplet hypothesis is calculated as:

$$\text{ABF} = \frac{1}{\sqrt{1+K}} \exp\left(-\frac{z^2}{2}\frac{K}{1+K}\right) \tag{S2}$$

where K represents the prior effect size under the alternative hypothesis, acting as a tuning parameter that controls the expected strength of the multiplet signal on the standardized z-score scale.

Once the frequentist evidence y is converted into the ABF, we integrate it directly with our existing snATAC-seq-derived posterior probability, $P(\text{multiplet} \mid \boldsymbol{x})$. By treating the ABF as the likelihood ratio for the new evidence, we modify our integration strategy (Equation 7 in main) to update the joint posterior probability as follows:

$$P(\text{multiplet} \mid \boldsymbol{x}, \boldsymbol{y}) \propto P(\text{multiplet} \mid \boldsymbol{x}) \cdot \text{ABF}_j(\boldsymbol{y}). \tag{S3}$$

This formulation allows us to rigorously update the probability of a droplet being a multiplet by weighting our HCLC-derived probability against the independent Bayesian evidence factor derived from the external modality.

3.3 Integrating Evidence from Existing Doublet Dection Methods

In this section, we list several commonly used multiplet detection methods, along with the data modalities they target and the types of evidence they produce as output.

Table S1: Summary of existing computational doublet-detection methods, their required data modalities, and the specific evidence types (scores) they generate.

Method	Data Modality	Evidence Type
**Scrublet**	scRNA-seq	**Likelihood Estimate**
**doubletCells**	scRNA-seq	**Enrichment Score**: Outputs a score defined as $p_A/(1-p_A)^2$, where p_A is the proportion of artificial doublets in its neighborhood.
**cxds**	scRNA-seq	**Sum of log p-values**: The score is the sum of negative natural log p-values of co-expressed gene pairs.
**bcds**	scRNA-seq	**Classification Probability**
**DoubletDetection**	scRNA-seq	**Average p-value**
**DoubletFinder**	scRNA-seq	**Enrichment Score**: The score is the proportion of artificial doublets among its k-nearest neighbors.
**Solo**	scRNA-seq	**Classification Probability**
**ArchR**	scATAC-seq	**p-values**
**AMULET**	scATAC-seq	**p-values**
**scDblFinder**	scRNA-seq & scATAC-seq	**Classification Probability**
**COMPOSITE**	scRNA-seq & scATAC-seq & ADT	**Posterior Probability**
**OmniDoublet**	scRNA-seq & scATAC-seq & ADT	**Classification Probability**

4 Computational Efficiency Benchmark

To evaluate the computational efficiency of SEBULA, we conducted a runtime and memory benchmark using three representative PBMC datasets (PB-1, PB-3, and PB-8). These datasets contain 13,093, 14,827, and 20,604 cells, respectively, with observed doublet proportions of approximately 21.3%, 22.8%, and 31.4%. Together, they represent moderate to relatively large single-cell experiments and span a range of doublet rates typical of droplet-based sequencing studies.

For fair comparison, we benchmarked the doublet detection stage of each method starting from its standard analysis-ready input. Specifically, SEBULA was timed beginning from the HCLC summary matrix (`colsum.csv`), which is generated after fragment-level preprocessing. ArchR was timed starting from pre-generated `Arrow files`, consistent with its recommended workflow. For scDblFinder applied to ATAC-seq data, timing was performed from the `SingleCellExperiment` object, as the feature aggregation step is internally coupled with the main doublet detection procedure and cannot be separated.

For each method, we recorded wall-clock runtime and peak resident memory usage during the detection step. All analyses were conducted in the same computing environment to ensure consistent resource measurement. The measured runtime and memory usage for each dataset and method are summarized in Table S2. The full benchmarking results are visualized in Supplementary Figure S3.

Table S2: Runtime and peak memory usage for doublet detection across three PBMC datasets. Runtime is reported in seconds and peak resident memory usage in gigabytes (GB).

Dataset	Method	Runtime (s)	Peak Memory (GB)
PB-1	SEBULA	1.75	0.165
PB-3	SEBULA	1.47	0.165
PB-8	SEBULA	2.10	0.167
PB-1	ArchR	622.51	6.42
PB-3	ArchR	646.39	6.93
PB-8	ArchR	985.88	10.27
PB-1	scDblFinder (ATAC)	187.98	14.06
PB-3	scDblFinder (ATAC)	212.72	16.16
PB-8	scDblFinder (ATAC)	337.83	23.93

5 Empirical Assessment of the Naïve Independence Assumption

The probabilistic integration framework used in SEBULA relies on a naïve Bayes formulation that assumes conditional independence between evidence sources given the multiplet status. Because violations of this assumption could potentially affect posterior confidence estimates, we performed an empirical assessment of the dependence between ATAC-derived and RNA-derived multiplet scores.

The analysis was conducted on the PB-3 dataset, which contains paired ATAC and RNA measurements. To focus specifically on the independence assumption under the null class, we restricted the analysis to cells annotated as singlets according to the experimental ground-truth labels. For each singlet cell, we obtained the ATAC-derived singlet probability from SEBULA and the RNA-derived singlet score from scDblFinder.

We quantified the association between these two evidence sources using both Pearson and Spearman correlation coefficients. Pearson correlation captures linear dependence between the scores, whereas Spearman correlation assesses monotonic relationships and is less sensitive to outliers.

Across the singlet population, the correlation between ATAC- and RNA-derived scores was modest (Pearson $r \approx 0.37$; Spearman $\rho \approx 0.13$). These values indicate limited redundancy between the two modalities among singlet cells. The relatively weak dependence suggests that the conditional independence approximation underlying the naïve Bayes integration is reasonable for the datasets considered. A scatter plot illustrating the relationship between the two scores is provided in Supplementary Figure S7.

References

1. Wolock SL, Lopez R, and Klein AM. Scrublet: computational identification of cell doublets in single-cell transcriptomic data. Cell systems 2019; 8:281–91

2. Wakefield J. A Bayesian measure of the probability of false discovery in genetic epidemiology studies. The American Journal of Human Genetics 2007; 81:208–27
