# Supplementary material for: Semi-parametric empirical bayes method for multiplet detection in snATAC-seq with probabilistic multi-omic integration
Source: PLoS Comput Biol. 2026 Apr 29;22(4):e1013653. doi: 10.1371/journal.pcbi.1013653 (PMC13148828; doi:10.1371/journal.pcbi.1013653)
Supplement: Attachment — Submitted filename: response.pdf [file pcbi.1013653.s009.pdf]

Response to Reviewers' Comments

Yuntian Wu, Alex Tsoi, and Xiaoquan Wen

We are grateful to all the reviewers for their insightful comments, helpful suggestions, and constructive criticisms. Following the suggestions of all three reviewers, we have made substantial revisions to the manuscript, with the Introduction and Discussion sections completely rewritten. In this document, we provide a point-by-point response to all reviewer comments. (The original reviewer comments are shown in blue font.)

Reviewer 1

1. It would be beneficial to explore and clearly articulate the limitations of the methods used in the study.

Response: We thank the reviewer for this suggestion. In response, we have revised the Discussion section and added a dedicated paragraph explicitly discussing the limitations of the proposed method (lines 267-279). We also discuss the potential violation of the naïve independence assumption in our evidence integration procedure and its practical implications (lines 259-266).

2. Consider including additional methodologies to enable a more comprehensive comparison and evaluation.

Response: We thank the reviewer for this suggestion. In the manuscript, we compare the

performance of SEBULA with other available multiplet detection methods from the following perspectives:

Comparison with methods using the same data feature For analyzing scATAC-seq data, our method is specifically designed to use the HCLC statistic derived from read-based fragment-level information. To the best of our knowledge, the most closely related approach using the same input is AMULET. Our synthetic data experiments therefore focus on comparing the computational performance of SEBULA and AMULET by simulating reads for artificial doublets and deriving the corresponding HCLC statistics. The improved performance of SEBULA over AMULET in this setting can be naturally attributed to the proposed modeling and computational framework.

Comparison with methods in real data applications Using the DOGMA-seq data, we benchmark additional multiplet detection methods, including scDblFinder, ArchR, and COMPOSITE. These additional comparisons serve two purposes:

1. When only ATAC-seq data are analyzed, we note that ArchR and scDblFinder utilize additional data features other than the HCLC statistic. Despite leveraging different sources of information, we find that SEBULA consistently outperforms AMULET and ArchR and performs comparably to scDblFinder. This suggests that HCLC is a powerful data feature for detecting multiplets, possibly due to its unique ability to detect homotypic multiplets.
2. We further find that the probabilistic integration framework implemented in SEBULA can effectively combine evidence from features both within and across data modalities, achieving even better performance than individual detection methods.

Based on the above experiments and observations, we acknowledge that methods leveraging data features beyond the ATAC-seq HCLC may provide additional evidence for detecting multiplets.

Nevertheless, because of SEBULA's unique ability to integrate evidence across methods and data modalities, we expect that the ensemble evidence produced by SEBULA is generally more comprehensive.

Following the reviewer's suggestion, we have added a more comprehensive review of existing multiplet detection methods in the Introduction (lines 62-81) and clarified the above points in the Discussion section (lines 250-256). Additionally, we have summarized some most popular multiplet detection methods in Table S1 of the Supplementary Material. The majority of the methods listed there can be combined with SEBULA.

3. A careful review of the manuscript for minor grammatical errors is recommended to ensure clarity and professionalism.

Response:

We thank the reviewer for this comment. We have made an effort to correct the grammatical errors in the original manuscript.

4. Improving the quality and resolution of the figures would significantly enhance the visual appeal and readability of the paper.

Response:

We thank the reviewer for this suggestion. We have regenerated the figures in the manuscript as vector graphics (EPS files) to ensure high resolution.

5. The manuscript would benefit from a more cohesive narrative structure. The abstract, introduction, and related work sections should clearly highlight the current challenges and gaps in the literature that the study aims to address.

Response: We appreciate the reviewer's thoughtful comment. Following the reviewer's suggestion, we have rewritten the abstract and Introduction to better highlight the current gap in the field

and the motivation for this work.

Reviewer 2

Major comments

1. The manuscript emphasizes the importance of calibrated probabilities over simple ranking. While the estimation of multiplet prevalence (π_0) is presented, the paper would greatly benefit from a more detailed analysis of the calibration itself. Reliability diagrams (calibration plots) comparing predicted probabilities to observed multiplet frequencies in the ground-truth datasets or quantitative scores such as expected calibration error could further strengthen the claim that SEBULA yields “well-calibrated posterior probabilities."

Response: We appreciate the reviewer’s comment. Following the reviewer’s suggestion, we conducted a more in-depth investigation of the calibration of posterior probabilities obtained from the nonparametric empirical Bayes model using simulated artificial doublet data.

We find that although the posterior probabilities exhibit some desirable calibration properties (e.g., reasonably accurate estimation of π_0, see Figure 3C and Supplementary Figure S2B) they do not satisfy a more stringent standard of calibration across the entire probability spectrum. More specifically, we observe that extremely high and low posterior probabilities tend to align well with their corresponding empirical frequencies, whereas intermediate posterior probability values show substantially poorer calibration.

To keep the focus of the paper on the classification performance of multiplet detection methods evaluated using rank-based metrics, we have removed statements claiming calibration of the output posterior probabilities. Nevertheless, having identified this issue, we plan to investigate it further in future work. We have also explicitly discussed this issue as a limitation of the current implementation in the Discussion section (Lines 272-279).

2. The method relies on the “zero assumption," specifically that the central mode of the data is dominated by singlets and that singlets are substantially more abundant than multiplets. While

this holds for standard experiments, "super-loaded" runs can have extremely high doublet rates. The authors could discuss or simulate the performance of SEBULA in scenarios with very high doublet rates to define the breaking point of this assumption. For example, demonstrations of failure modes and guidance for identifying them would strengthen reader confidence.

Response: We thank the reviewer for raising this important point and agree with the reviewer's assessment regarding the importance of the "zero assumption." In practice, doublet rates typically range from 5-30% in standard single-cell experiments, depending on the loading concentration, and our simulation experiments adopt settings consistent with this practical range. More precisely, as we now discuss in the Discussion section (Lines 238-242), our method only requires singlets to dominate the low-HCLC region (i.e., the observed low-HCLC peak is primarily contributed by singlets), which is a weaker condition than requiring singlets to dominate multiplets in the overall dataset.

Following the reviewer's suggestion, we performed additional stress-test simulations mimicking "super-loaded" doublet scenarios. The newly added Figure S6 shows the distribution of the transformed HCLC data under a simulation setting with an approximately 40% doublet rate (the different colors of the histograms represent singlets and doublets, respectively). The figure suggests that the zero assumption continues to hold well under this setting. SEBULA subsequently estimates the singlet proportion as $\pi_0 = 0.608$, which is reasonably close to the true value of 60%. More generally, we observe that the mode representing singlets remains consistently present even when the simulated doublets substantially outnumber singlets (lines 246-247). Based on these additional experiments, we conclude that the zero assumption is robust in most practical settings.

Additionally, we note the following:

1. Extremely high multiplet rates are uncommon in practice. Most droplet-based protocols are optimized to balance throughput and multiplet rates, and very high multiplet fractions substantially degrade data quality and are therefore generally avoided.

2. SEBULA includes built-in numerical and visual diagnostics that help detect potential violations of model assumptions, including violations of the zero assumption.

3. The current validation focuses heavily on benchmarking metrics (F1 scores, ROC curves). To increase the relevance of the manuscript, it would be beneficial to demonstrate the impact of using SEBULA on a downstream biological application or something similar. Additionally, the authors could explicitly discuss the limitation that the method requires fragment files and cannot run on processed count matrices alone, which may limit its applicability for some users.

Response: We thank the reviewer for this thoughtful comment. Our validation scheme is primarily motivated by the fact that multiplet detection and removal are commonly used as important quality control (QC) and preprocessing steps in single-cell data analysis. Accordingly, our benchmarking metrics focus on evaluating classification performance using both simulated and real datasets with carefully annotated ground truth.

In light of the reviewer's comment, we have revised the manuscript to clarify this motivation and added additional citations highlighting the negative effects of multiplets on downstream analyses, particularly their potential harm in generating spurious biological findings (lines 40-55).

The reviewer is also correct that SEBULA, by design, relies only on fragment-level information to detect multiplets in ATAC-seq data. This design choice is motivated by the fact that fragment files are typically available and required for standard ATAC-seq quality control and analysis workflows, including peak calling and the computation of commonly used QC metrics such as TSS enrichment and nucleosome signal. However, existing methods have not fully leveraged fragment-level information for multiplet detection. This point is illustrated in our DOGMA-seq experiments, where SEBULA, using only fragment-level information, achieves performance comparable to or better than computational tools that rely on additional sources of information in ATAC-seq analysis. Additionally, SEBULA's probabilistic evidence integration framework is designed to address the reviewer's concern: the probabilistic evidence derived from fragment-level

information can be naturally combined with quantitative evidence derived from other features and modalities of single-cell data to achieve even better detection performance.

Following the reviewer's suggestion, we now have explicitly discussed the reliance on fragment files as a computational limitation of the current implementation of SEBULA (lines 267-271).

4. The multimodal integration uses a naïve Bayes assumption. Although the authors note the limitations, the performance comparisons against COMPOSITE may not be fully fair unless the independence assumption is examined. An analysis when naïve Bayes fusion may inflate or deflate confidence due to dependence would be valuable.

Response: We thank the reviewer for raising this important point. We agree that the naïve independence assumption employed by the naïve Bayes framework can be overly strong and unrealistic for real data. This general issue is widely acknowledged in the machine learning literature and may partially contribute to the lack of calibration in the reported posterior probabilities.

Nevertheless, the consensus in the field recognizes that even when the naïve independence assumption is not strictly satisfied, the model can still achieve good classification performance because the resulting decision boundaries may be relatively insensitive to inaccuracies in the estimated class-specific densities ([1], p. 346, and also [2, 3, 4]). As we explained in our previous response, our evaluation of SEBULA focuses primarily on its classification performance, and we indeed observe this phenomenon predicted by theory in both our simulation and real data experiments.

Additionally, we examined the correlation between ATAC- and RNA-derived scores within the singlet population using the PB-3 dataset. We observed only weak correlations (Pearson $r = 0.37$, Spearman $\rho = 0.13$), suggesting limited dependence between the two modalities among singlets (Supplementary Figure S7). Thus, we conclude that the naïve independence assumption is not severely violated in this setting.

We have added this new analysis, along with the corresponding discussion, to the revised manuscript (lines 256-266) and Supplementary Materials Section 5.

5. Figure 1 is intended to provide an overview of the framework, but it currently lacks context regarding the problem setup. The figure should ideally illustrate the data acquisition or the biological origin of multiplets to set the stage. A "zoom-in" approach detailing the different stages (preprocessing vs. detection vs. integration) would make the workflow easier to parse visually.

Response: We greatly appreciate the reviewer's comments. We have completely recreated Figure 1 according to the reviewer's excellent suggestions.

6. Can the authors comment on further literature and their relevancy such as OmniDoublet (and the references in there such as Scrublet and DoubletFinder)?

Response: We thank the reviewer for the suggestion. We have revised the manuscript to discuss the referenced methods (lines 62-81; lines 87-89; lines 252-256), focusing on their methodological contributions to multiplet detection and their potential to be combined with SEBULA.

Briefly, we view OmniDoublet [5] as a significant advancement in multimodal doublet detection. It integrates transcriptomic and epigenomic signals through Jaccard similarity-based weighting, thereby improving detection accuracy and robustness by leveraging complementary information across modalities in multimodal single-cell datasets.

OmniDoublet, as well as Scrublet [6] and DoubletFinder [7], builds upon the key idea of bootstrapping the doublet distribution by generating artificial doublets from the observed data. This framework has proven highly effective, particularly for transcriptomic data and for detecting heterotypic doublets in datasets with well-resolved cellular clusters. Nevertheless, recent studies suggest potential limitations of such approaches. In particular, doublets in real single-cell data may deviate from the simple additive behavior of singlets due to technical and biological factors, including variable capture efficiency and the decline effect (i.e., reduced per-cell contribution when

multiple nuclei occupy the same droplet) [8]. We observed a similar phenomenon in our data when comparing simulated doublets with ground-truth doublets using the HCLC feature, where the two distributions exhibit noticeable differences. As a result, simulated doublets may not fully recapitulate the distribution of true multiplets, which could affect the subsequent classification performance.

Finally, we believe that SEBULA and OmniDoublet (or other simulation-based doublet detection methods) can truly complement each other through the proposed probabilistic evidence integration framework. By combining evidence derived from different data features and distinct computational principles, the resulting ensemble evidence can potentially achieve “the best of both worlds" and further improve multiplet detection performance.

Minor comments

1. The introduction establishes the problem well, but the focus on the integration of multiple modalities appears somewhat late. Given that SEBULA-MI (the integrated version) yields the best performance, this motivation should be highlighted earlier in the introduction to better frame the paper's contributions.

We thank the reviewer for this excellent suggestion. We have revised the Introduction section (lines 82-91) to emphasize the motivation for multi-modal integration earlier in the narrative.

2. Detecting homogeneous doublets is rather difficult in general, what would be the impact on a downstream analysis of missing them?

We thank the reviewer for this comment. In many standard single-cell workflows, homotypic doublets are often considered relatively innocuous ([9]). However, their presence can affect certain downstream analyses. For example, Thibodeau et al. [10] emphasize that filtering homotypic multiplets is important for analyses involving allelic bias and lineage tracing, where aggregated fragment counts can distort per-cell allele-specific or lineage signals. Similarly, McGinnis et al.

[7] note that the presence of doublets may alter the structure of dropout patterns, potentially biasing imputation methods that rely on transcriptional similarity.

We have added the relevant discussion to the revised Introduction section (lines 45-52).

3. The Box–Cox transformation is a key component of the pipeline, yet several choices are presented as default without a clear rationale. The transformation may blur biologically meaningful differences across cells with extreme HCLC.

We thank the reviewer for raising this important point. In the revised manuscript, we have clarified the rationale for using the Box-Cox transformation and the choice of default settings.

We agree with the reviewer that the Box-Cox transformation may alter the quantitative differences among cells. However, as a monotonic transformation, the Box-Cox function preserves the rank ordering of the cells, which makes it possible to better separate the singlet and doublet distributions.

4. The abbreviation "SEBULA" is introduced without a clear explanation of what the acronym stands for in the main text (it appears to be derived from the title, but this should be explicit).

We thank the reviewer for pointing this out. In the revised manuscript, we now explicitly define the abbreviation “SEBULA” at its first occurrence in the main text, clarifying that it stands for **S**emi-parametric **E**mpirical **B**ayes approach for m**UL**tiplet detection in single-nucleus **A**TAC-seq.

5. In Figure 2, it is unclear which panels correspond to labels A and B. Moreover, the text claims the singlet distribution is "multimodal". However, in the density plot (Figure 2A), the singlet distribution appears as a single sharp spike. If multimodality exists, the x-axis scale or visualization needs to be adjusted to make this visible to the reader.

We thank the reviewer for this comment. We have substantially updated Figure 2 in the revised manuscript.

6. Runtime comparisons against the other methods would highlight SEBULA's practical usability at scale.

We thank the reviewer for this suggestion. In the revised manuscript, we have added runtime benchmarking results and now summarize the computational performance comparison in the Results (Lines 221-226), with additional details provided in Supplementary Materials Section 4 and Supplementary Figure S3. We also note in the Discussion that preprocessing the fragment files to generate HCLC statistics remains the main computational bottleneck in the current implementation (Lines 268-271).

7. A few minor spelling errors, e.g., "applyed," (l.172) "effectively" (l.168), should be corrected.

We thank the reviewer for this comment. We have corrected the typos in the revised manuscript.

Reviewer 3

Major:

1. The method uses a truncation threshold, x_{min}. Although an optimization procedure (minimizing cross-entropy) is provided and the Methods section claims robustness, including a sensitivity analysis (e.g., a plot of F1 scores as x_{min} varies) would better demonstrate SEBULA's robustness.

Response: We thank the reviewer for this excellent comment. Following the reviewer's suggestion, we have added the requested sensitivity analysis in this revision. Specifically, the newly added Supplementary Figure S5 shows the impact of varying x_{min} (from 0 to 11) on multiple classification performance metrics. We observe that the optimal F1 scores remain practically invariant, while AUROC and AUPRC vary only minimally.

We have also revised the Methods section (lines 350-355) to explicitly reference these sensitivity analyses, supporting our claim that performance is stable across a broad range of x_{min} values. In the software documentation, we have also added explicit descriptions of the sensitivity analysis procedures.

2. HCLC (High-coverage loci) is defined according to AMULET's preprocessing. As HCLC is a critically important concept to the SEBULA method, it would be helpful to briefly clarify whether this preprocessing step is computationally expensive or if SEBULA includes an optimized preprocessor, as this step is often the bottleneck in read-based methods.

Response: We thank the reviewer for this comment. We directly adopt the HCLC preprocessing algorithm established in AMULET and reimplement a pre-processor as an R function in SEBULA, which takes the compressed fragment file as input. We find that our implementation scales well for large single-cell datasets. For the PB-3 dataset (16,550 cells; input fragment file size 2.6 GB), the full preprocessing required to generate the HCLC statistic takes 1,725 seconds (28.8 minutes) of wall-clock time, with a peak memory usage of 0.11 GB on a Linux workstation (Intel Xeon

Gold 6148, 80 logical CPUs, 1.0 TB RAM). We acknowledge that the reviewer's assessment is correct: compared with the downstream analysis steps, the HCLC preprocessing stage is indeed the most computationally intensive component of the SEBULA pipeline.

In the revised manuscript, we now clarify the preprocessing steps used to extract the HCLC statistic in the Methods section (Lines 281-295). We also added discussion in the Discussion section to note that, although SEBULA is efficient once the HCLC statistics are provided, extracting these statistics from ATAC-seq fragment files can be computationally expensive and remains the main practical bottleneck of the current pipeline (Lines 268-271).

3. The integration of ATAC and RNA signals relies on a "naïve Bayes" assumption of conditional independence between the modalities. While the authors acknowledge this is an approximation, the manuscript would benefit from a brief analysis or plot (perhaps in Supplementary Materials) showing the correlation between HCLC scores and RNA-based doublet scores within the singlet population. If the correlation is low, this would empirically justify the independence assumption.

Response: We thank the reviewer for this excellent suggestion. In response, we examined the correlation between ATAC-derived HCLC scores and RNA-based doublet scores within the singlet population using the PB-3 dataset. We observed relatively weak correlation between the ATAC- and RNA-derived signals among singlets (Pearson $r \approx 0.37$, Spearman $\rho \approx 0.13$), which suggests that the naïve independence assumption is not severely violated. Following the reviewer's suggestion, we have added this analysis to the Supplementary Materials Section 5 and added corresponding figure (Supplementary Figure S7) and discuss its implication in the main text (lines 256-266).

More broadly, the naïve independence assumption is known to be a strong modeling assumption. Nevertheless, existing literature [1, 2, 3, 4] shows that classification performance can remain robust to violations of this assumption (even though the resulting posterior probabilities may become uncalibrated). Please also refer to our response to Comment 4 from Reviewer 2 for

additional discussion.

Minor

1. Although the authors claim the software is "computationally efficient," the Results section lacks specific data to support this. Including a brief comparison of runtime and memory usage between SEBULA, AMULET, and ArchR on a representative dataset would be beneficial.

Response: We thank the reviewer for this suggestion. In the revised manuscript, we added computational benchmarking results to better document SEBULA's practical performance on large-scale single-cell datasets. These runtime comparisons are now summarized in the Results section (lines 221-226), with additional benchmarking details provided in the Supplementary Materials Section 4 and corresponding figure (Supplementary Figures S3). We also note that AMULET has a computational cost similar to that of SEBULA, because the computational bottleneck for both methods is the HCLC preprocessing step (lines 268-271), as correctly pointed it out by the reviewer in the previous comment.

2. Figure 1 shows that SEBULA can integrate ATAC and RNA data. However, it is unclear whether SEBULA supports integration of more than two modalities, as newer sequencing technologies like DOGMA-seq and TEA-seq profile ATAC, RNA, and surface protein. If so, the Methods section and Figure 1 should be updated accordingly.

Response: We thank the reviewer for this comment. Following the reviewer's suggestion, we have updated the Methods section and Figure 1 to clarify that SEBULA-MI can accommodate more than two modalities.

3. Rank-preserving Box-Cox transformation: this transformation always preserves ranks as long as λ is fixed. It is unclear why the term "rank preserving" needs to be emphasized.

The reviewer is correct. We have removed the term "rank preserving".

4. I noticed several typos:

Page 12, line 172: "applyed" should be "applied".

Page 12, line 174: "profiles" should be "profiling".

Page 12, line 189: "effectivey" should be "effective".

Page 16, line 245: "provieds" should be "provides".

Response: We thank the reviewer. We have corrected the typos.

References

1. Bishop CM and Bishop H. Deep learning: Foundations and concepts. Springer Nature, 2023
2. Domingos P and Pazzani M. On the optimality of the simple Bayesian classifier under zero-one loss. Machine learning 1997; 29:103–30
3. Rish I et al. An empirical study of the naive Bayes classifier. *IJCAI 2001 workshop on empirical methods in artificial intelligence*. Vol. 3. 22. Seattle, USA. 2001 :41–6
4. Zhang H. The optimality of Naive Bayes. *Proceedings of the Seventeenth International Florida AI Research Society Conference, FLAIRS 2004*. AAAI Press, 2004 :562–7
5. Liu L, Ren J, Zhou X, Cheng X, Pan X, Zhou L, Lu Y, and Liu P. OmniDoublet: a method for doublet detection in multimodal single-cell sequencing data. Briefings in Bioinformatics 2025; 26:bbaf538
6. Wolock SL, Lopez R, and Klein AM. Scrublet: computational identification of cell doublets in single-cell transcriptomic data. Cell systems 2019; 8:281–91
7. McGinnis CS, Murrow LM, and Gartner ZJ. DoubletFinder: doublet detection in single-cell RNA sequencing data using artificial nearest neighbors. Cell systems 2019; 8:329–37
8. Hu H, Wang X, Feng S, Xu Z, Liu J, Heidrich-O'Hare E, Chen Y, Yue M, Zeng L, Rong Z, et al. A unified model-based framework for doublet or multiplet detection in single-cell multiomics data. Nature Communications 2024; 15:5562
9. Xi NM and Li JJ. Benchmarking computational doublet-detection methods for single-cell RNA sequencing data. Cell systems 2021; 12:176–94
10. Thibodeau A, Eroglu A, McGinnis CS, Lawlor N, Nehar-Belaid D, Kursawe R, Marches R, Conrad DN, Kuchel GA, Gartner ZJ, et al. AMULET: a novel read count-based method for effective multiplet detection from single nucleus ATAC-seq data. Genome biology 2021; 22:1–19
